# Single-molecule visualization of dynamic transitions of pore-forming peptides among multiple transmembrane positions

**DOI:** 10.1038/ncomms12906

**Published:** 2016-09-30

**Authors:** Ying Li, Zhenyu Qian, Li Ma, Shuxin Hu, Daguan Nong, Chunhua Xu, Fangfu Ye, Ying Lu, Guanghong Wei, Ming Li

**Affiliations:** 1 Beijing National Laboratory for Condensed Matter Physics and CAS Key Laboratory of Soft Matter Physics, Institute of Physics, Chinese Academy of Sciences, Beijing 100190, China; 2 State Key Laboratory of Surface Physics, Key Laboratory for Computational Physical Sciences (Ministry of Education), and Department of Physics, Fudan University, Shanghai 200433, China; 3 School of Physical Sciences, University of Chinese Academy of Sciences, Beijing 100049, China

## Abstract

Research on the dynamics of single-membrane proteins remains underdeveloped due to the lack of proper approaches that can probe in real time the protein's insertion depth in lipid bilayers. Here we report a single-molecule visualization method to track both vertical insertion and lateral diffusion of membrane proteins in supported lipid bilayers by exploiting the surface-induced fluorescence attenuation (SIFA) of fluorophores. The attenuation follows a *d*^−4^ dependency, where *d* is the fluorophore-to-surface distance. The method is validated by observing the antimicrobial peptide LL-37 to transfer among five transmembrane positions: the surface, the upper leaflet, the centre, the lower leaflet and the bottom of the lipid bilayer. These results demonstrate the power of SIFA to study protein-membrane interactions and provide unprecedented in-depth understanding of molecular mechanisms of the insertion and translocation of membrane proteins.

## Abstract (web summary)

Assessing protein localization within lipid membranes is problematic. Here, the authors describe a single molecule visualization method based on surface-induced fluorescence attenuation (SIFA) to determine the insertion depth and lateral diffusion of a peptide in a lipid bilayer.

## Graphical abstract

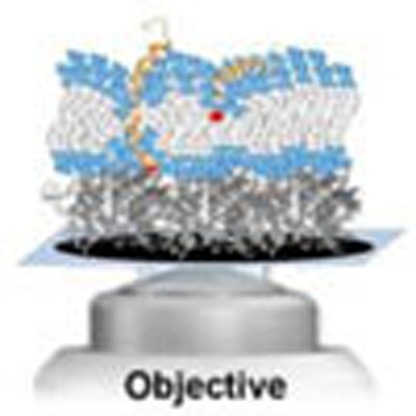

It is estimated that more than a third of all genes encode membrane proteins^1^. Knowledge about the orientation and depth of insertion of these proteins in biological membranes is crucial to understanding their functions^2^. In general, probes that yield such information should have a nanometre or even sub-nanometre precision since the thickness of the membranes is usually only about 4 nm. The probes should also be able to distinguish the protein's vertical insertion from the lateral diffusion that arises from the fluidity of the membranes. For studying protein–membrane interactions, an additional criterion should also be born in mind: fewer disturbance to the membrane is better. Single-molecule fluorescence approaches are biologists' favourite choice because they can be visualized and readily incorporated into super-resolution microscopy. However, the lack of proper tools that can probe the transmembrane positions of fluorophores in lipid bilayers with nanometre resolution has hindered the study of protein–membrane interactions. Here we report a single-molecule imaging method to visualize three-dimensional (3D) motions of membrane proteins in supported lipid bilayers, taking advantage of the surface-induced fluorescence attenuation (SIFA) of single emitting fluorophores^3^,^4^,^5^,^6^. The technique is analogous to the fluorescence resonance energy transfer (FRET) exept that in SIFA, energy is transferred from a point to a plane while in FRET, energy is transferred from a point to another point.

Although not very effective, a few conventional techniques have already been used to study protein–membrane interactions. FRET can be used to probe nano-scale displacements of dye-labelled proteins^7^,^8^,^9^. Although it is able to detect single molecules, this point-to-point technique usually does not distinguish lateral motion from vertical one, and is useful only for a few special cases, where the positions of acceptors are precisely known. Environment-sensitive dyes provide information about the polarity surrounding dyes^10^,^11^, but do not yield more once the dyes are already inside the membrane. Fluorescence quenching by brominated lipids has nanometre sensitivity^12^,^13^, but suffers from the drawback that it does not distinguish the states in the two leaflets of the bilayer. All these techniques would fail if the lipid bilayers were seriously disturbed. For instance, when toroidal pores are formed in the presence of antimicrobial peptides, the transmembrane position of the peptides cannot be derived from the environment-sensitive dyes, nor can it be derived from the fluorescence quenching by brominated lipids, because the toroidal pores are composed of peptides intercalated with lipids. The peptides in such situations sense the same lipidic environment as when they lie parallel to the surface of the membrane, contributing no specific signal to the techniques using environment-sensitive dyes or brominated lipids.

We applied SIFA to study the pore-forming antimicrobial peptide LL-37 and demonstrate the validity of this technique to track single fluorophores in bilayers. Antimicrobial peptides can selectively kill bacterial cells and are hence considered as the future therapeutic agents as more bacterial strains become resistant to conventional antibiotics^14^,^15^,^16^,^17^. LL-37 is involved in diverse biological processes such as immunomodulation, apoptosis, angiogenesis and wound healing. Previous studies showed that LL-37 induces local defects and forms multimeric toroidal pores in lipid bilayers when the peptide:lipid ratio exceeded a threshold value^14^,^16^,^17^. Despite intensive investigations, details of the pore-formation dynamics of LL-37 are still elusive. A recent sum frequency generation (SFG) vibrational spectroscopic assay revealed that the inserted LL-37 can slip out of the bilayer, leading to a parallel orientation of the peptide^18^. The ensemble measurement, however, did not give a clue about how this takes place at the molecular scale. Membrane conductance studies on single pores revealed that melittin and other antimicrobial peptides induce transient pores that do not exhibit well-defined single-channel step conductance, indicating the formation of multi-level pores^19^,^20^. What is the structural basis of the multi-level pore and whether LL-37 has a similar behaviour remain to be investigated^21^. SIFA enabled us to measure the transmembrane positions of the dye-labelled LL-37 with nanometre precision, revealing the existence of five transmembrane states of the peptide.

## Results

### Nanometre sensitivity of SIFA

The physical basis of SIFA is the strong electromagnetic coupling between a fluorescing molecule and an absorbing surface^3^,^4^,^5^. The fluorescence attenuation follows a quenching equation,


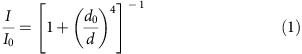


where *I*_0_ is the fluorescence intensity of a free fluorophore, *I* the intensity near the surface and *d*_0_ the characteristic quenching distance at which *I*/*I*_0_=1/2. For studying proteins in lipid bilayers, a good choice of *d*_0_ is ∼4 nm. To find a proper attenuating surface, we tested a few materials including ultra-thin gold films, indium-tin oxide (ITO) glasses and graphene oxide (GO) layers. It turned out that the characteristic quenching distances of gold and ITO are too large (tens of nanometres) to yield acceptable signal to noise ratios. Even worse, surface roughnesses of metal layers or ITO may weaken the SIFA effect because of the locally enhanced electric field on rough surfaces^22^. Moreover, the roughness may result in uncertainty in the intensity-to-distance conversion. GO stands out because of being transparent, inherently flat and with adjustable quenching distance via ultra violet irradiation^6^,^23^,^24^. It is worth noting that, as in single-molecule FRET assays, we do not use SIFA to measure the absolute distance between the fluorophore and the surface. Instead, we measure the relative change in position of the fluorophore in the bilayer. From a practical perspective, one can use a parameter d*, which is the distance between the top of the supported bilayer under study and the GO layer, to replace *d*_0_. Starting from the quenching equation above, we obtained the following equation for the relative position after some algebra,


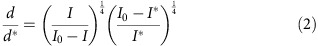


where I* is the fluorescence intensity of the fluorophore on top of the supported bilayer under study. Intensity fluctuations therefore dominate the relative precision of *d*.

**Figure 1:**
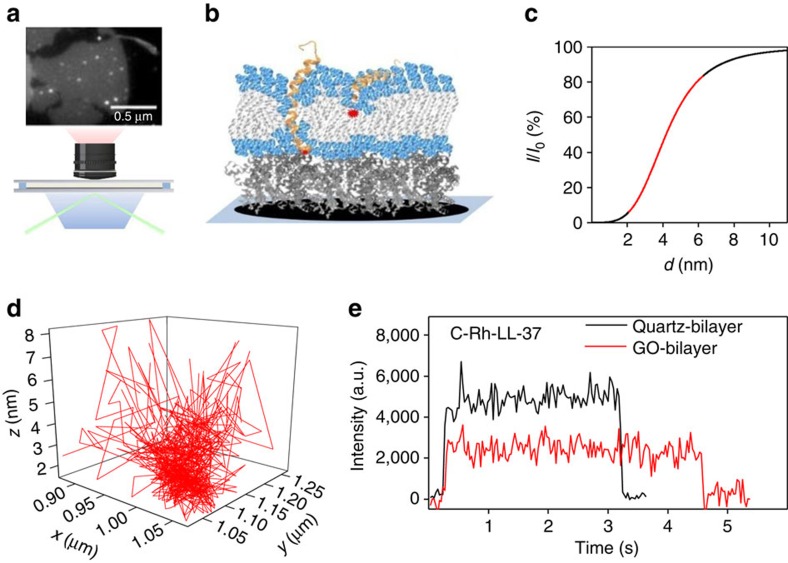
The surface-induced fluorescence attenuation (SIFA) method. (**a**) The experimental setup. Inset on the top is an image of N-Rh-LL-37 on a GO-supported bilayer. The grey sheet is the GO layer. (**b**) Sketch of a lipid bilayer lifted by a cushion on top of a GO layer. The yellow helices represent the peptides that can insert in the bilayer with different depths. (**c**) The degree of attenuation of a dye as a function of the dye-surface distance. The red segment on the curve represents the most sensitive range of SIFA. (**d**) 3D trace of a N-Rh-LL-37 molecule in the supported DMPG bilayer. (**e**) Comparison of the fluorescent intensity of a C-Rh-LL-37 molecule on a GO-supported bilayer (red) to that on a quartz-supported bilayer (black).

In this work, we used GO monolayers deposited on glass surface as the absorber and used a TIRF microscope to track both the vertical insertion and the lateral diffusion of single-membrane proteins in supported bilayers (Fig. 1a,b). The experimental procedures are outlined as follows, with details presented in Methods and Supplementary Information. We deposited a GO layer on a quartz slice^25^ and glued together the slice with a coverslip to make a flow chamber. We then produced a dimyristoyl-phosphatidylglycerol (DMPG) bilayer on the GO layer following a liposome fusion method^26^. We checked the stability and the homogeneity of the bilayer by imaging the bilayer doped with fluorescent lipids. We also checked the fluidity of the bilayer by fluorescence recovery after photobleaching. We labelled LL-37 with rhodamine at the N-terminus (N-Rh-LL-37) or C-terminus (C-Rh-LL-37), mixed the labelled LL-37 with unlabelled ones, and then injected the mixture into the flow chamber. Unless specified elsewhere, the ratio of the labelled to the unlabelled LL-37 is set to be 1:100 (with a final concentration of the labelled peptides being 20 nM) in order to avoid trajectory overlapping in a single pore (that is, to lower the possibility of having two or more dyes in a single pore). Shown in Fig. 1d, is a 3D trace of the N-terminus of an LL-37 molecule in a supported bilayer (Fig. 1b). The in-plane position (the *xy* coordinate) was read from the images, and the transmembrane position (the *z* coordinates) was derived from the intensity, *I*(*x*,*y*), of the fluorophore according to the quenching equation. The lateral motion and the vertical insertion of the proteins were hence decoupled in our approach, which is important because the membrane proteins should be allowed to diffuse freely in the membrane to mimic the natural conditions. It is seen that the range of motion of a molecule in the *xy* plane is larger near the surface than near the bottom, which is understandable because Brownian motion is hindered when the molecule is inside the bilayer, especially when it is in an oligomer.

The nanometre sensitivity of SIFA to distance change is demonstrated by comparing the fluorescent intensities of C-Rh-LL-37 molecules on a GO-supported bilayer, a GO-supported BSA monolayer and a quartz-supported bilayer (Fig. 1e and Supplementary Fig. 1). The C-terminus of LL-37 stays at the surface of the bilayer. The fluorescence intensities are, respectively, 48±6%, 31±6% and 100±6% (mean±s.d.) of that of a rhodamine molecule on the glass surface. According to the thicknesses of the lipid bilayer (∼4.1 nm)^27^ and the self-assembled monolayer of the BSA proteins (∼3.3 nm)^28^, the characteristic quenching distance is derived to be *d*_0_=4.0±0.5 nm for the GO monolayers used in the present work. The value is close to the one given by Huang *et al*.^29^.

### A single LL-37 may insert in the lipid bilayer transiently

**Figure 2:**
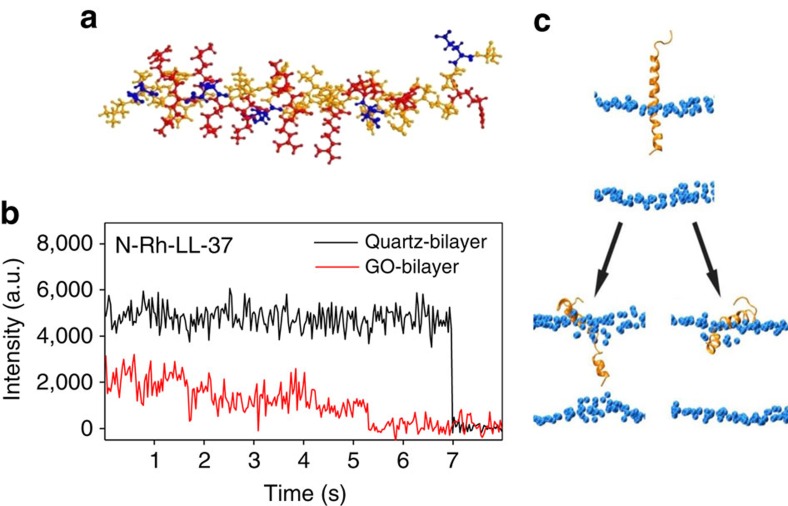
Insertion of a single N-Rh-LL-37 in the bilayer. (**a**) The structure of LL-37 (PDB ID: 2K6O). Red: basic residues. Blue: acidic residues. Orange: uncharged residues. (**b**) Comparison of the fluorescent intensity of an N-Rh-LL-37 molecule on GO-supported bilayer (red) to that on quartz-supported bilayer (black). (**c**) Atomstic MD simulations of an LL-37 molecule in a DMPG bilayer using the Gromos87-based force field. Top: initial configuration where the LL-37 monomer was partially inserted in the bilayer; Lower left: the peptide inserts deeply into the lower leaflet; Lower right: the peptide returns closer to the upper surface.

The fluorescent behaviour of the N-Rh-LL-37 molecules on GO-supported lipid bilayers is more interesting. When the surface density of the peptides was very low, most of the N-Rh-LL-37 molecules behaved similarly to the C-Rh-LL-37 molecules. A small proportion of them showed significant fluorescence fluctuations (Fig. 2b and Supplementary Fig. 2). This unexpected enhancement in fluorescence fluctuations should be attributed to the variation of the distance of the fluorophore to the GO layer. It might result from the distortion of the bilayer by the peptide, which was reported to be 1–2 nm (refs 30, 31), or the transient insertion of the LL-37 molecule in the lipid bilayer^32^. The former could be excluded because the fluorescence of C-Rh-LL-37 did not exhibit similar fluctuations.

To confirm the membrane insertion capability of an LL-37 monomer at the molecular level, we performed multiple atomistic molecular dynamics (MD) simulations using two different atomistic force fields, with each simulation started from a state in which an LL-37 monomer was partially-inserted in a DMPG bilayer (Fig. 2c and Supplementary Fig. 5). The reason that we chose this partial-insertion state as the starting configuration of the MD simulations is given in Supplementary Information. In two out of four MD runs, the LL-37 monomer inserted deeply in the lower leaflet of the lipid bilayer with the N-terminal residue *Leu*1 contacting with the phosphorus atoms at the lower surface (Fig. 2c, bottom left and Supplementary Fig. 5a,b). In the other two runs, the peptide moved towards the upper surface with the N-terminal residues being embedded at the head–tail interface of the upper leaflet and the helical residues from 2 to 31 being nearly parallel to the surface (Fig. 2c, bottom right and Supplementary Fig. 5a,c). Detailed analysis on the number of hydrogen bonds and the interaction energy between LL-37 and lipid molecules (Supplementary Fig. 6) showed that the electrostatic interactions play an important role in peptide insertion, while the hydrogen bonds help stabilize the membrane-bound configuration.

### LL-37 moves up and down at high surface density

**Figure 3:**
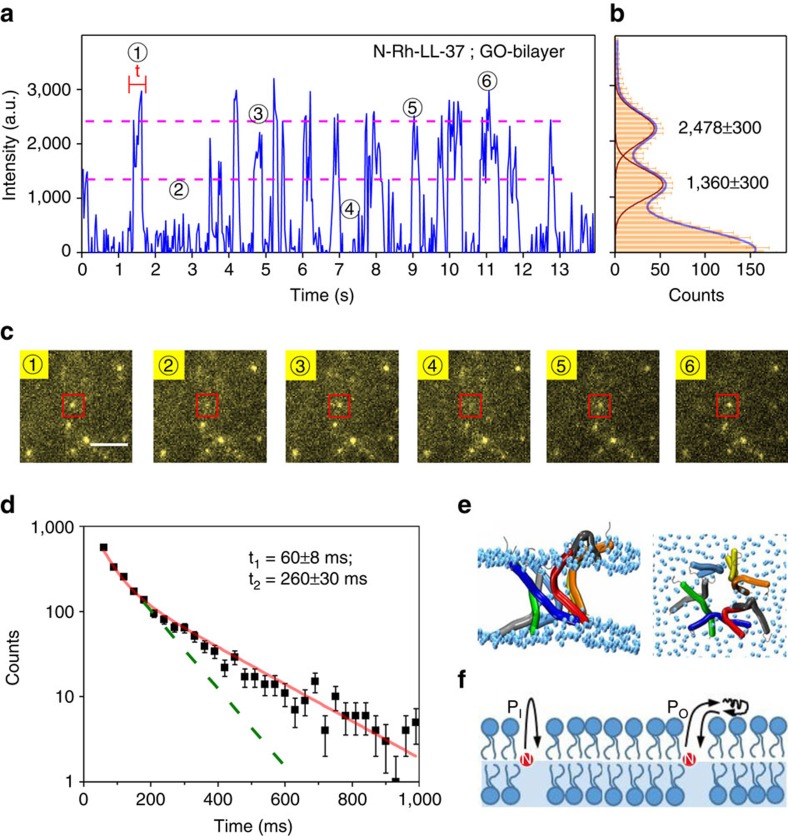
Dynamics of N-Rh-LL-37 in the bilayer. (**a**) A fluorescence trace of an N-Rh-LL-37 molecule on a GO-supported DMPG bilayer. (**b**) Probability distribution function (PDF) of the fluorescent intensity built from five typical traces. (**c**) A few images corresponding to the makers in the trace. Scale bar, 5 μm. (**d**) PDF of the dwell time of the high fluorescence states. The definition of the dwell time is indicated by a red bar in (**a**). (**e**) Side and top views of the final state of a representative 1-μs-long coarse-grained MD simulation. The peptides are shown in bendix representation and the phosphorus atoms in light blue spheres. (**f**) Two possible pathways of LL-37 in the bilayer. P_I_: the inner pathway with LL-37 moving inside the bilayer; P_O_: the out pathway with LL-37 slipping out of the surface and then back. The error bars denote standard deviation.

The LL-37 peptides induce pores in lipid bilayers when the surface density of LL-37 is high enough^16^,^17^,^18^,^21^,^33^,^34^,^35^,^36^. Atomic force microscopy (AFM) showed that accumulation of LL-37 at bilayers composed of anionic PG (phosphatidyl-DL-glycerol)/dPG (di-PG) (95:5 w/w) induces individual wormholes or toroidal pores^35^. Neutron scattering revealed that LL-37 forms toroidal pores in fully hydrated DOPC bilayers^21^. An LL-37 monomer has a net charge of +6 at neutral pH. The positively charged LL-37 adsorbs more readily onto anionic lipid bilayers^17^,^36^. Although we could not see pores with the SIFA technique, we inclined to believe that pores could form in our systems because the experimental conditions are very similar to those under which many antimicrobial peptides induce toroidal pores in bilayers^17^,^21^,^33^,^34^,^35^,^36^. We observed that LL-37 accumulated on the bilayer over time due to the electrostatic attraction between the cationic peptides and the anionic lipids (Supplementary Fig. 3a). We waited for about 1 h so that a large number of pores could form. As a result, the amount of the fluorophores on the bilayer became too dense to be resolved individually. To facilitate single-molecule observation, we applied continuous laser illumination to bleach most of the fluorophores (Supplementary Fig. 3c). The remaining ones were recorded for data analysis. They could be basically divided into two classes. A large proportion of them stayed on the surface of the bilayer. They were bright but were bleached out very soon. Less than 15% of the N-Rh-LL-37 molecules were less mobile and could stand the laser irradiation for longer than 5 min. Surprisingly, the fluorophores in this class flickered repeatedly and three fluorescence levels were recorded (Fig. 3a). The phenomenon was not observed for N-Rh-LL-37 on quartz-supported bilayers. Neither was it observed for C-Rh-LL-37 on GO-supported bilayers (Fig. 1e). The flickering of the fluorescence means that the N-Rh-LL-37 molecule moves up and down in the bilayer repetitively.

We built the probability density function (PDF) (Fig. 3b) of the fluorescent intensities by simply collecting all points in five typical traces, one of which is in Fig. 3a. There are two peaks in the PDF beside the background. We fitted the PDF with two Gaussians and converted the peak values to transmembrane positions, which are at 3.1±0.4 (mean±s.d.) nm and 4.0±0.4 nm above the GO layer, respectively. Fluorophores with the lowest intensities are in the ‘blind' region of SIFA because the fluorescence is strongly attenuated and becomes undistinguishable from the background noise if the fluorophores are at <2 nm above the GO layer (either in the lower leaflet or on the bottom of the bilayer) (Fig. 1c). We built a PDF of dwell times of the high fluorescence states (see Fig. 3a for the definition of the dwell time). The PDF can be fitted with a double exponential function with decay constants of 60±8 ms and 260±30 ms, respectively (Fig. 3d). We believe that the two time constants correspond to two kinetic processes.

To understand the dynamics of LL-37, we performed 20 independent 1-μs-long coarse-grained MD simulations of toroidal pores formed by eight LL-37 monomers each. It turned out that the pores are thermodynamically stable although the peptides in some pores (∼20%) may slip out of the pores in some MD runs. A simulation snapshot in Fig. 3e shows that a peptide slipped out of the pore and stayed parallel to the membrane surface. The out-slipped peptide may return to its original position (Supplementary Fig. 7), indicating that the LL-37 peptides composing the toroidal pores are dynamic. We also performed four independent 1-μs-long MD simulations of toroidal pores formed by 10 LL-37 monomers each. Similar results were obtained. We speculate that the fast (60 ms) kinetics corresponds to the peptide moving up and down inside the pore, while the slow (260 ms) kinetics corresponds to the peptide having reached the membrane surface before it moving back (Fig. 3f). The simulations also showed that the pore size reduces when a peptide slips out of the pore. The reduction of the pore size does not cause the pore to disrupt. Our results are consistent with those for melittin and other antimicrobial peptide pores whose sizes were also shown to vary with time by membrane conductance measurements of single pores^19^,^20^. Moreover, the transfer of the N-terminus to the surface confirms the recent SFG result that inserted LL-37 can be pulled out of the membrane^18^. We also performed a 350-ns atomistic MD simulation in order to examine the structural stability of a more realistic LL-37 pore in the DMPG bilayer. Our simulation showed that the LL-37 pore maintained during the full period atomistic MD simulation. The surrounding lipids tilted as the simulation time increased and a toroidal pore was observed at *t*=350 ns (Supplementary Fig. 8). However, due to the large size of the system, it is computationally challenging to observe the up and down movement of the peptide in the DMPG bilayer. This is also the main reason that we carried out multiple long-time scale coarse-grained MD simulations.

### Transfer of the peptide among five transmembrane positions

**Figure 4:**
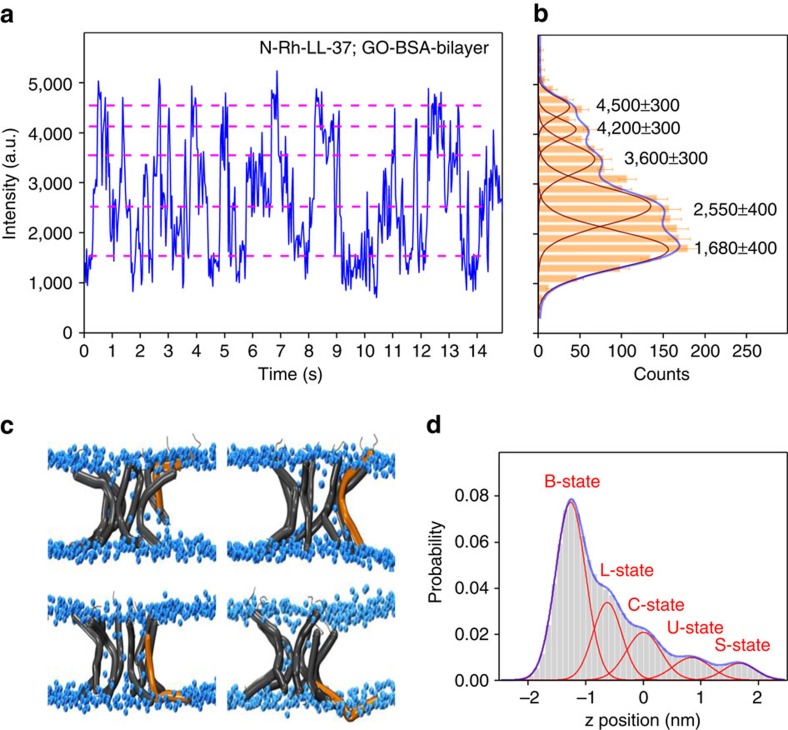
Transition of an N-Rh-LL-37 molecule among five transmembrane positions. (**a**) Fluorescence trace of an N-Rh-LL-37 molecule in a GO-BSA-supported bilayer. (**b**) PDF of the fluorescent intensity built from 4 typical traces. (**c**) Snapshots of toroidal pores from four 1-μs-long coarse-grained MD simulations. (**d**) PDF of the transmembrane position of the *Leu*1 residue according to the MD simulations. The error bars denote standard deviation.

To solve the ‘blind' region problem of SIFA, we deposited a layer of BSA proteins (∼3.3 nm thick) on the GO layer to shift the lipid bilayer out of the ‘blind' region (Fig. 1b). The fluorescence traces now contain much more information about the transmembrane positions of the peptides. It is faintly visible in Fig. 4a that the peptide prefers to stay at a few levels (dashed lines). We built a PDF of the intensities by collecting all points in four typical traces like that in Fig. 4a and then made statistical analysis. A few peaks that correlate with the levels in the traces are faintly visible (Fig. 4b). In addition, our coarse-grained MD simulations indicated that the peptides in the pores prefer to stay at a few transmembrane positions (Figs 3e and 4c). We hence tried to fit the PDF to a function with a few Gaussian peaks, each peak corresponding to a preferred position of the peptide in the bilayer. We set the number of Gaussian peaks *N* to be a free parameter in the fitting process. It turned out that *N*=5 resulted in the best fit. It is worth noting that a fit with *N*=4 was almost as good as the one with *N*=5 if the PDF of intensity was analysed only. The uncertainty arises from the fact that the top surface of the bilayer is ∼7 nm above the GO layer, already in the saturation region of the attenuation curve in Fig. 1c. Fortunately, two factors can be used to improve the analysis. (1) The topmost two dashed lines in Fig. 4a suggest that there are two states near the saturation region. (2) The two peaks in Fig. 3b also indicate the existence of two states in the upper leaflet of the bilayer. We therefore took *N*=5 in the subsequent analysis and got five transmembrane positions at 3.4±0.4 (mean±s.d.), 4.2±0.4, 5.2±0.4, 6.2±0.4 and 7.5±0.9 nm above the GO layer, respectively. The positions correspond to the bottom surface, the lower leaflet, the centre, the upper leaflet and the top surface of the lipid bilayer, respectively. Our measurements hence revealed that the peptides in the pores are at thermodynamic equilibrium, moving up and down in the pores. To the best of our knowledge, this is the first direct single-molecule observation of the dynamics of pore-forming peptides in biological membranes.

The LL-37 peptides in the 20 1-μs-long MD runs experienced the transmembrane positions revealed by SIFA. This argument is confirmed by the theoretical PDF of the transmembrane positions of the N-terminal residue *Leu*1 derived from the MD data (Fig. 4d), which can also be fitted with five Gaussian peaks. To compare the SIFA results with the MD simulations, we converted firstly the fluorescence intensity-versus-time traces into the position-versus-time traces according to equation (2) and built the PDF of positions from four such traces (Supplementary Fig. 4). The two PDFs agree well with each other except that the probability density of the bottom state in the SIFA PDF is slightly lower than that in the MD PDF. The discrepancy may arise from the fact that the BSA cushion underneath the bilayer may hinder the motion of the LL-37 to the bottom surface of the bilayer. Therefore, both the SIFA and the MD results suggested that the N-terminus of LL-37 transfers among five transmembrane positions and prefers to insert deeply in the membrane. We calculated the interaction energy between the dynamic LL-37 and the lipid molecules surrounding it, using the molecular configurations given by MD. It turned out that the peptide–lipid interaction energy has a similar PDF no matter it stays on the upper leaflet, the centre or the lower leaflet of the bilayer (Supplementary Fig. 9a). However, the PDF of the peptide-peptide interaction energy showed that the peptide prefers a deeper insertion because the interactions in the lower leaflet are significantly stronger than those in the upper leaflet and the centre (Supplementary Fig. 9b). This is understandable because the LL-37 peptides in the toroidal pore are intercalated with lipids, sensing similar lipidic environment when they move up and down in the pore. It is the peptide–peptide interactions that dictate LL-37 to prefer the lower leaflet of the bilayer.

**Figure 5:**
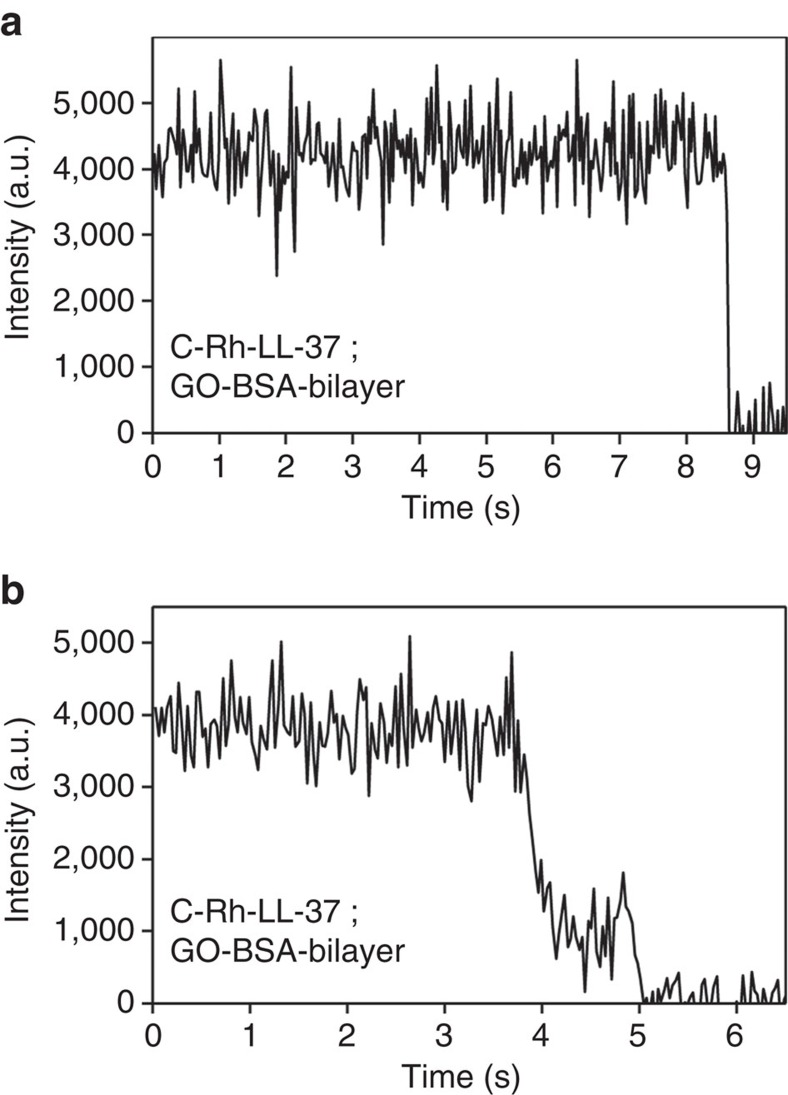
Control experiments with C-Rh-LL-37 in the GO-BSA-supported bilayer. (**a**) More than 94% of the fluorescence traces are flat. (**b**) A very small fraction of the traces exhibit fluctuations.

We did a few control experiments with LL-37 labelled at the C-terminus (C-Rh-LL-37), which gave very different results under the same experimental conditions. For example, we showed in Fig. 1e a typical trace of C-Rh-LL-37 on a GO-supported bilayer. One sees that the fluorescence intensity is flat before the fluorophore quenches. The intensity traces became a little different when the C-Rh-LL-37 molecules were on the GO-BSA-supported bilayer. Most traces resemble that in Fig. 1e. A very small fraction (<6%) of them exhibit weak fluctuations (Fig. 5), suggesting that the C-terminus of LL-37 can also be buried in the bilayer. However, the mean intensity is higher than that of the N-Rh-LL-37, indicating that the C-terminus of LL-37 does not insert in the bilayer as deeply as the N-terminus. The results are in accordance with our MD simulations that the C-terminus of an LL-37 molecule in a pore could be pulled into the bilayer when the N-terminus moves out to the bottom surface (Fig. 4c). In addtion, we performed similar experiments at temperatures below 20 °C, which is lower than the main transition temperature of the lipid, and did not observe any strong flickerring of the N-Rh-LL-37 molecules.

## Discussion

**Figure 6:**
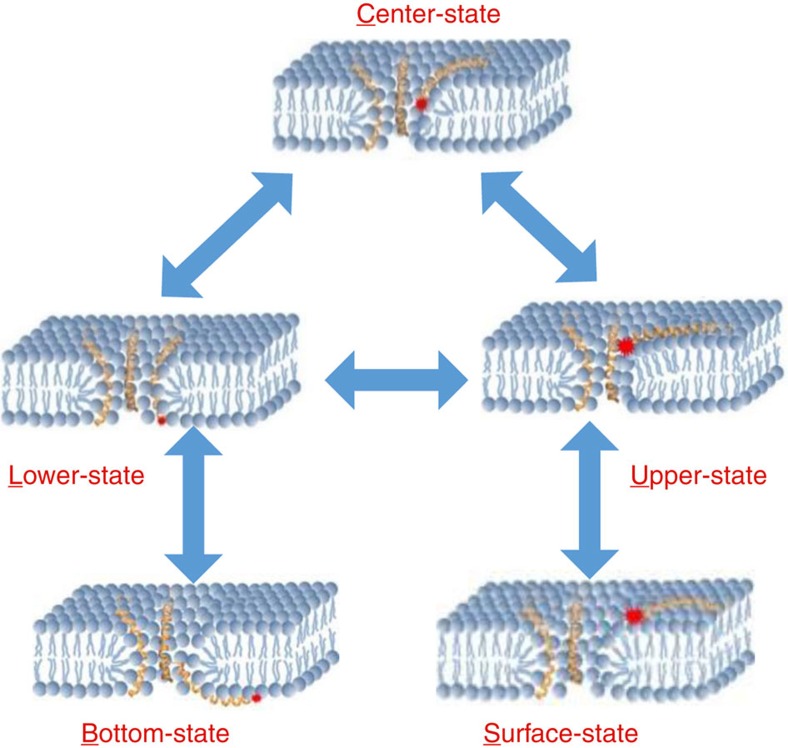
Model of the dynamic toroidal pore of LL-37. The LL-37 monomer transfers among five transmembrane positions: the surface, the upper leaflet, the centre, the lower leaflet and the bottom.

We demonstrated that SIFA is a powerful tool to track the insertion depth of single-membrane proteins/peptides and to distinguish which part of a protein/peptide inserts how deeply into the supported lipid bilayer. We applied the SIFA method to study the pore-forming peptide LL-37. The results suggested that the pores induced by LL-37 are dynamic and the LL-37 monomers in the pores transfer dynamically among five transmembrane positions: the surface, the upper leaflet, the centre, the lower leaflet and the bottom (Fig. 6). The ability to distinguish five transmembrane positions in a space as thin as ∼4 nm indicates that SIFA has an unprecedented precision for the studies of protein-membrane interactions. On the other hand, our results suggested that the dynamics of the toroidal pores is unexpectedly sophisticated. The interplay between the experimental observations and the theoretical simulations provided a deep insight into the dynamics of the antimicrobial peptides.

The key to a successful SIFA measurement is to tune the characteristic quenching distance, *d*_0_, so that the motion of the fluorophores is in the most sensitive range of detection. We tuned the quenching distance of GO to ∼4 nm so that the brightness of a fluorophore changes from 90 to 25% of its intrinsic value as the dye-labelled protein moves from the top to the bottom of a lifted lipid bilayer. The quenching distance of a completely reduced GO layer can be significantly increased^37^. SIFA can therefore be extended to thicker systems. For example, it may be applied to study how membrane proteins travel across the inter-membrane space of a double-membrane system such as those in mitochondria and bacteria. The protein dynamics in the double-membrane systems was even less studied than in the single-membrane systems. We believe that SIFA will find more applications in the research of membrane active systems.

## Methods

### The LL-37 peptides

The human cathelicidin LL-37 has 37-residues, LLGDF FRKSK EKIGK EFKRI VQRIK DFLRN LVPRT ES. The N-terminal rhodamine labelled LL-37 (N-Rh-LL-37), C-terminal rhodamine labelled LL-37 (C-Rh-LL-37) and unlabelled LL-37 were all synthesized by China Peptides Co. Ltd. (Shanghai, China). The antimicrobial activity of the labelled LL-37 was tested following the protocol reported in the literature^38^. *Escherichia coli* MG1655 was used in the test. The overnight culture of the bacterial was diluted 1:500 into 500 μl Luria broth medium. The unlabelled LL-37, N-Rh-LL-37, C-Rh-LL-37 of 5 μl, all with a concentration of 4 μM, were added to samples 1, 2 and 3, respectively. Sample 4 was used as a control. The cells were incubated in a 37 °C shaker (Thermo Forma 481) at 250 r.p.m. for 12 h. The optical density at 600 nm was measured using a spectrometer (NanoDrop 2000c) before and after the incubation. The labelled LL-37 has nearly the same antimicrobial activity as the unlabelled one. The cell growth was inhibited completely at the concentration 4 μM (Supplementary Fig. 10).

### Sample chambers

The fabrication of GO followed the modified Hummers method^39^,^40^. The GO layers were deposited on plasma-treated quartz by using the Langmuir–Blodgett technique. A GO-covered quartz slice and a clean coverslip were glued together with double-sided tapes (50 μm thick, 3M Corp) to make the flow chamber. The sealed chamber was treated with ultraviolet irradiation (365 nm, 48 W) for longer than one hour to reduce the auto-fluorescence of the GO layers^24^. The duration of the ultraviolet irradiation was set by balancing the demanded characteristic quenching distance *d*_0_ and the remaining auto-fluorescence of GO. The flow chamber was filled with MilliQ water before use.

### GO-supported lipid bilayers

Approximately 200 μl MilliQ water was flowed into the chamber to soak the GO layers^26^; 100 μl sonicated suspension of DMPG vesicles was then flowed slowly into the chamber and incubated at 50 °C for 2–3 h, followed by cooling for 30 min (the lowest temperature should be higher than the main transition temperature of lipids). The aggregates were washed away by replacing the solution in the chamber with PBS buffer for 10–20 times (200–300 μl each time). We usually used the chamber immediately after the bilayer preparation.

### GO-BSA-supported lipid bilayers

Following a reported procedure^28^, tetraethelepentamine (TEPA) was dissolved in deionized water to a final concentration of 75 mM and pH=5.1 ml of 25-fold diluted TEPA solution was mixed thoroughly with 100 μl BSA solution (1 mM). After the addition of 1 μl fresh EDC (50 mM) and 1 μl fresh NHS (100 mM), the solution was incubated at room temperature for 10 h with vigorous shaking (250 r.p.m.). Production of 200 μl was flowed slowly into the sample chamber (50 μl min^−1^). Another 200 μl solution was added after 30 min. To wash away the extra solution, 2 ml deionized water was used for 2–3 times followed by 400 μl PBS buffer. The DMPG bilayer was then prepared as before.

### Experimental procedures

Since the main purpose of the experiments is to demonstrate the validity of SIFA in tracking single dyes, we mixed the labelled LL-37 with unlabelled ones (1:100) in order to avoid trajectory overlapping in the same pore. The total concentration of LL-37 is 2 μM. This strategy is in accordance with the general rule that the optimal labelling densities must balance the need for high-density sampling of molecular behaviour against practical requirements for accurate and unbiased single-molecule detection and tracking^41^. The LL-37 solution was flowed into the sample chamber. After incubation for about 1 h, the fluorescence was recorded by a TIRF microscope equipped with an EMCCD. The regions of interest (ROIs) were selected by ImageJ. A Spot Tracker plug-in was used for the position tracking and intensity recording of each spot.

### Characteristic quenching distance of the GO layers

SIFA is not sensitive to distance changes outside the 0.5 *d*_0_–1.7 *d*_0_ range. An appropriate *d*_0_ should therefore be selected so that the sensitive range of SIFA covers more than a half of the thickness of the membrane. Although a value of *d*_0_ <3 nm results in a higher precision, the sensitive range is too small to be able to cover the upper leaflet of the bilayer. A value of 3–6 nm is the optimum choice for *d*_0_. Two reference layers on top of which the dyes were located were used to estimate the characteristic quenching distance *d*_0_ of the GO layers. One was the GO-supported lipid bilayer (DMPG lipid bilayer). The other was GO-supported BSA monolayer. When the C-terminal labelled LL-37 molecules were added to the reference system, their fluorescence intensity is attenuated. The measurements were performed under the same conditions with the same batch of the labelled peptides (Supplementary Fig. 1).

### Prism-based TIRF imaging

The flow chamber was imaged with a prism-based TIRF microscope. The optical setup follows the traditional prism-type TIRF microscope^42^. The prism was placed on top of a quartz slide of the flow chamber with a thin layer of immersion oil (Olympus, *n*=1.518) in between. The microscope is equipped with a 532-nm laser (Compass 315M, Coherent). The fluorescence collected by an oil-immersion objective (× 100, NA=1.45, Olympus) was selected by a dichromatic mirror (Semrock 577/20) and an emission filter (Semrock 610/30) and recorded by an electron-multiplying charge-coupled device (EMCCD) camera (iXon 897, Andor Technology) with a frame rate of 30 frames per second. The images were recorded using the Solis image capture software (Andor). The regions of interest (ROI, usually 7 × 7 pixels) were selected by ImageJ. A Spot Tracker plug-in was used for the position tracking and intensity recording of each spot.

### Molecular dynamics (MD) simulations

The MD simulations were performed with the GROMACS software package^43^ in a combination with the MARTINI coarse-grained model (for both proteins and lipids^44^,^45^ and the atomistic model (Gromos 87 force field for proteins and Berger force field for lipids).

We performed four independent 100-ns atomistic MD simulations started from a configuration where the LL-37 monomer was partially inserted in DMPG lipid bilayer. This strategy allowed us to examine whether LL-37 preferentially binds to the membrane surface or stays inside the bilayer within the time scales accessible in atomistic MD simulations.

We performed twenty 1-μs coarse-grained (CG) MD simulations for the 8-mer LL-37 toroidal pore and four 1-μs CG-MD simulations for the 10-mer pore in DMPG lipid bilayer (Supplementary Tables 1 and 2). An 8-membered pore model (inner radius≈3.2 nm) is built with the hydrophilic face of LL-37 exposed to water, and placed into the DMPG bilayer by the program INFLATEGRO from Tieleman's group^46^ with lipids inside the pore removed. Counterions (Na^+^) are added to neutralize the system. This system is energy minimized and solvated with water, followed by energy minimization and a protein-position-restrained simulation for 100 ns, to obtain a well-equilibrated toroidal pore. In this work, the peptide:lipid ratio is 8:458. We also carry out MD simulations in other peptide:lipid ratios, and find that a stable toroidal structure cannot exist when the number of peptides is less than six.

We also performed one 350-ns atomistic MD simulation in order to probe the structural stability of a more realistic LL-37 pore in DMPG bilayer. The simulation started from an octamer in which each of the eight peptide chain is in an α-helical structure and perpendicular to the normal of the DMPG bilayer. Similar to the CG-MD simulations, the peptide:lipid ratio is 8:458. There are 127,509 atoms in this atomistic LL-37 octamer-membrane system. It can be seen from Supplementary Fig. 8 that the LL-37 pore remained stable during the full period MD simulation and it gradually changed into a toroidal shape. In addition, each LL-37 peptide kept α-helical conformation.

### Data availability

The data that support the findings of this study are available from the corresponding author on request.

## Additional information

**How to cite this article:** Li, Y. *et al*. Single-molecule visualization of dynamic transitions of pore-forming peptides among multiple transmembrane positions. *Nat. Commun.* 7:12906 doi: 10.1038/ncomms12906 (2016).

## Supplementary Material

Supplementary Information Supplementary Figures 1-10, Supplementary Tables 1-2, Supplementary Methods, Supplementary References
