# Supplementary material for: Single-molecule visualization of dynamic transitions of pore-forming peptides among multiple transmembrane positions
Source: Nat Commun. 2016 Sep 30;7:12906. doi: 10.1038/ncomms12906 (PMC5056435; doi:10.1038/ncomms12906)
Supplement: Supplementary Information — Supplementary Figures 1-10, Supplementary Tables 1-2, Supplementary Methods, Supplementary References [file ncomms12906-s1.pdf]

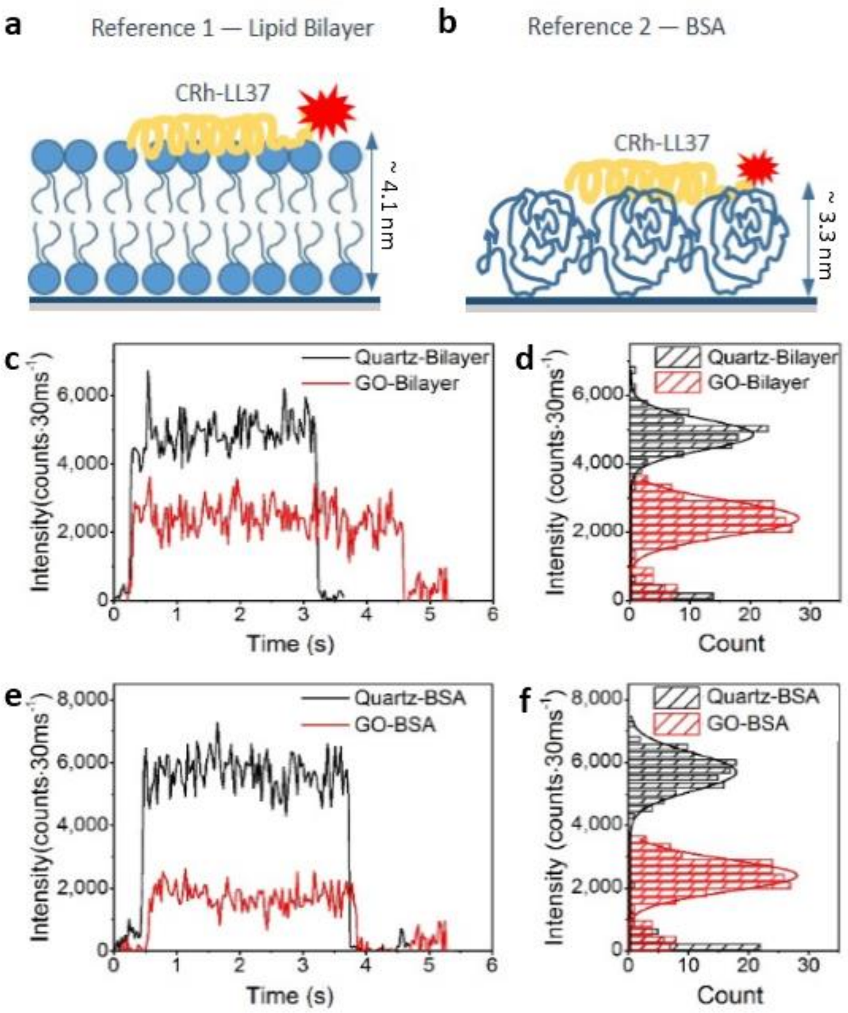

Supplementary Figure 1. The SIFA effect of the GO layer on the C-Rh-LL-37 molecules. (**a**) Schematic illustration of a C-Rh-LL-37 molecule on top of a GO-supported lipid bilayer. (**b**) A C-Rh-LL-37 molecule on the BSA protein monolayer (~3.3 nm thick). (**c**) Comparison of the fluorescence intensity of the C-Rh-LL-37 on the GO-supported bilayer (red) with that on the quartz-supported bilayer (black). The corresponding intensity distributions are shown in (**d**). (**e**) Comparison of the fluorescence intensity of the C-Rh-LL-37 on the GO-supported BSA monolayer (red) with that on the quartz-supported BSA monolayer (black). The corresponding intensity distributions are shown in (**f**).

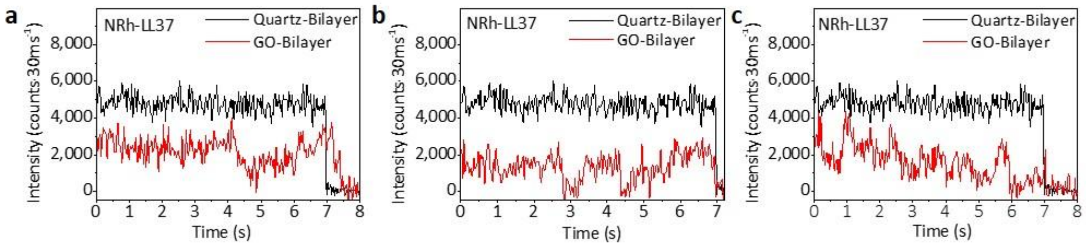

Supplementary Figure 2. Fluorescence traces of N-Rh-LL-37 molecules on GO-supported bilayer at low surface density. The large intensity fluctuations of the red curves correspond to 1~2 nm variation of the dye-to-surface distance.

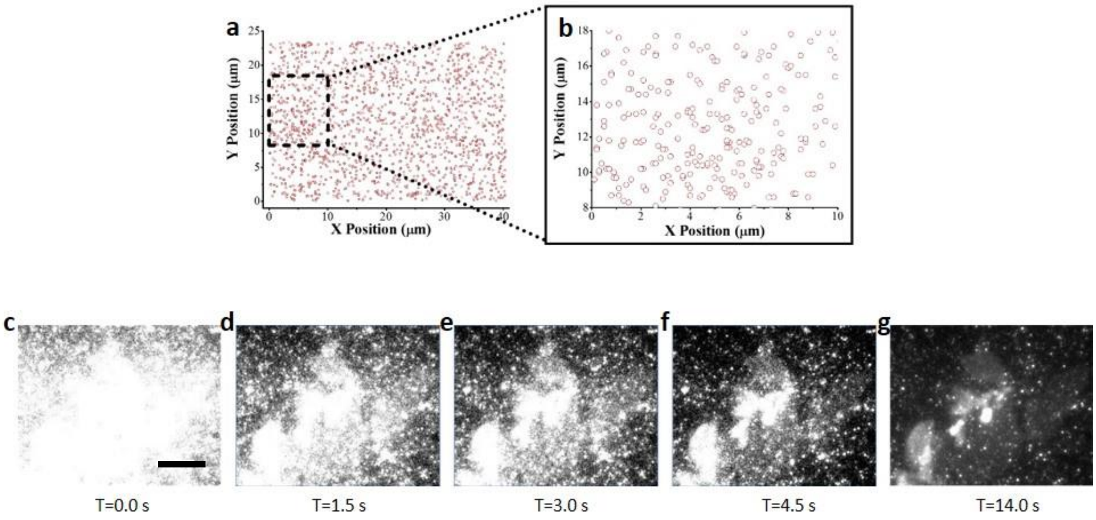

Supplementary Figure 3. The accumulation of LL-37 and the continuous illumination induced bleaching. (**a-b**) Accumulation (~15 min) of the LL-37 molecules on the lipid bilayer. The number of the Rh-LL-37 molecules on the bilayer increases with time. After about one hour, the amount of the dyes on the lipid bilayer becomes too dense to be resolved individually. (**c-g**) Fluorescence images of N-Rh-LL-37 on GO-supported bilayer after laser illumination of 0-14 seconds. Continuous laser illumination bleaches most of the dyes eventually. Scale bar is 10 μm.

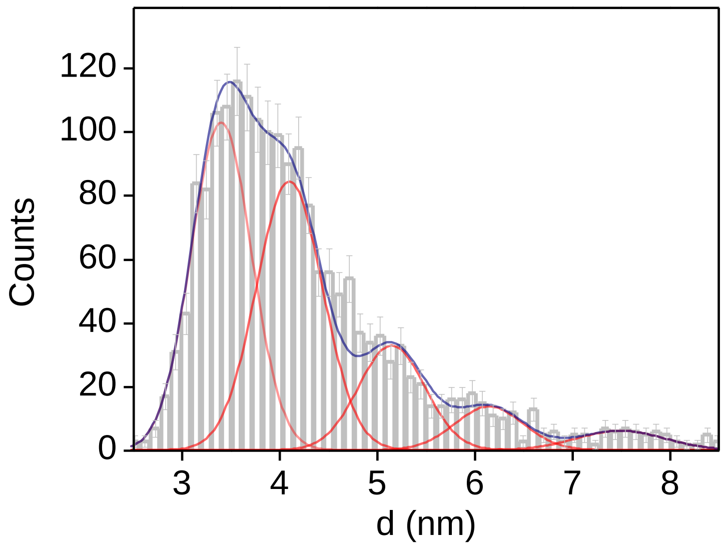

Supplementary Figure 4. Probability distribution function (PDF) of transmembrane positions of the N-terminus of LL-37 in GO-BSA-bilayers. This PDF corresponds to the PDF in Fig. 4b in the main text. In order to compare the SIFA results with the MD simulations, we converted firstly the fluorescence intensity-vs-time traces into the position-vs-time traces according to Eq. (2) and built the PDF.

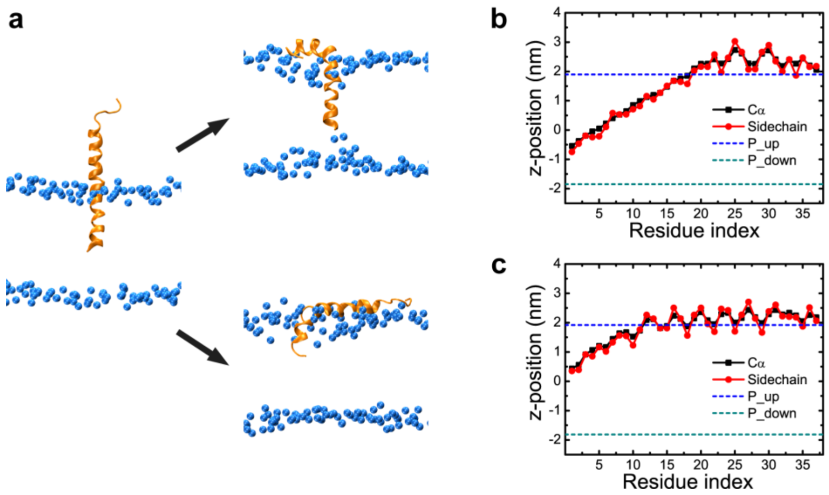

Supplementary Figure 5. Analysis of the interaction between an LL-37 monomer and the DMPG lipid bilayer. The results were obtained from two representative 100-ns MD simulations using the GROMOS53A6/GROMOS-CKP force field. (**a**) Snapshots of an LL-37 molecule in a DMPG lipid bilayer: the initial configuration where the LL-37 monomer is partially-inserted in a DMPG lipid bilayer (left) and two different final states (right) where the LL-37 peptide is mostly buried in the bilayer (upper) and binds to the surface of the upper leaflet (lower). The transmembrane positions of the Cα atom (black) and the side chain centroid (red) of each amino acid residue for the LL-37 monomer (**b**) buried in the lower leaflet of the bilayer and (**c**) binding to the surface of upper leaflet of the bilayer.

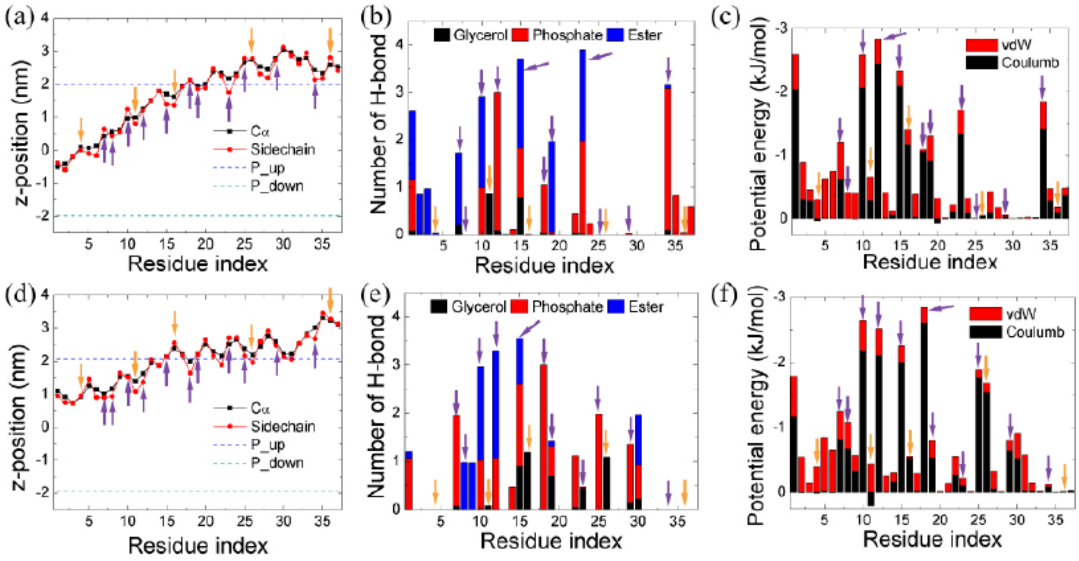

Supplementary Figure 6. Analysis of the interaction between an LL-37 monomer and the DMPG lipid bilayer. The results were obtained from two representative 100-ns MD simulations using the GROMOS87 and modified Berger force fields. (a) and (d) The transmembrane positions of the Cα atom (black) and the side chain centroid (red) of each amino acid residue. (b) and (e) Number of H-bonds formed between each amino acid residue and three different groups in the lipid heads: glycerol (black), phosphate (red), and ester (blue) groups. (c) and (f) The potential energy of each individual residue with the DMPG bilayer (per lipid): the electrostatic (black) and vdW (red) component. The LL-37 monomer inserts more deeply into the lower leaflet of the bilayer in two out of four 100-ns MD runs (a-c), different from the results of the other two runs (d-f), where the peptide move back to the surface of the upper leafet. Each point is an average of the last 20 ns of two independent 100-ns MD runs. The purple and orange arrows point to the positively and negatively charged residues, respectively.

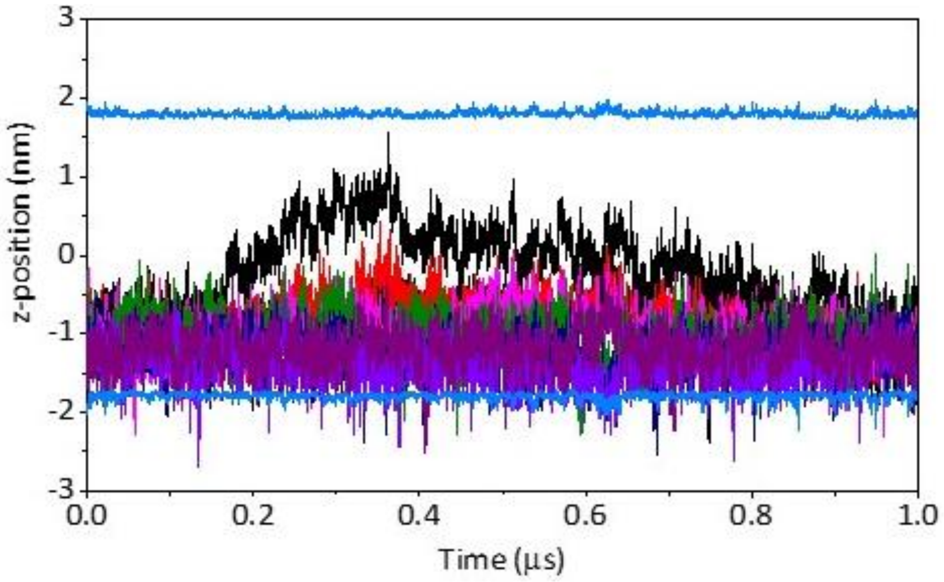

Supplementary Figure 7. Representative MD traces of the transmembrane position (z-position) of the *Leu*1 residue (the first residue of LL-37). The different colors correspond to the different peptide chains in the toroidal pore. The blue lines correspond to the position of phosphorus atoms at the top and the bottom surfaces of the bilayer. A peptide (black line) climbs off the pore at t=0.16 μs and returns to the original position at t=0.8 μs.

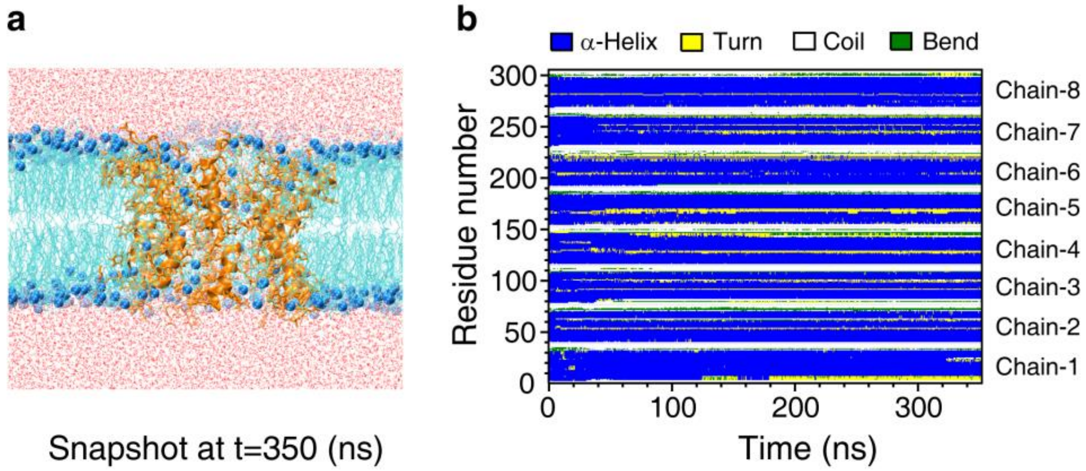

Supplementary Figure 8. Atomistic simulation of an LL-37 octameric pore in DMPG bilayer. This simulation is 350-ns long and it started from an octamer in which each of the eight peptide chain is in an α-helical structure and perpendicular to the normal of the DMPG bilayer. **(a)** The snapshot at t=350 ns, where a toroid pore is observed. (**b)** Time evolution of the secondary structure of LL-37 octamer. The peptide main chain is shown in cartoon representation, with the positively charged residues in blue, the negatively charged residues in red, the hydrophilic residues in green, and the hydrophobic residues in white. Bond representation is given for the sidechain of each amino acid residue. The phosphorus atoms are shown in tan spheres and lipids are not shown for clarity. The LL-37 octamer-membrane system contains 127509 atoms.

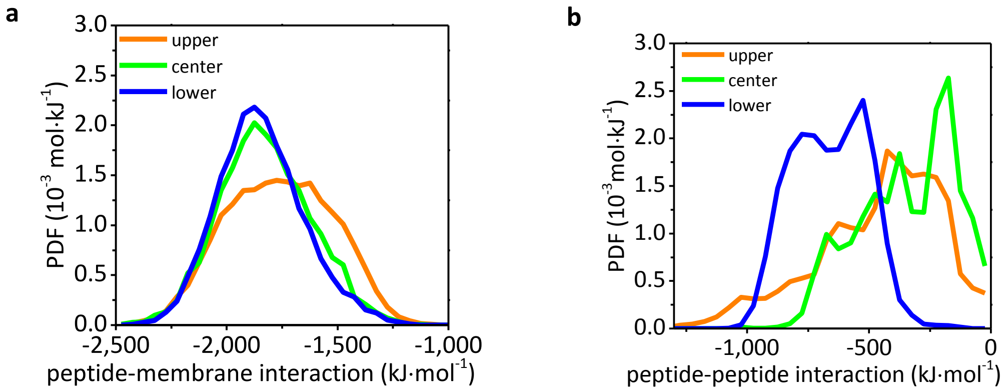

Supplementary Figure 9. The peptide-membrane and the peptide-peptide interaction energies. (**a**) Probability density function (PDF) of the peptide-lipid interaction energy at the lower leaflet, the center and the upper leaflet states. (**b**) PDF of the peptide-peptide interaction energy at the lower leaflet, the center and the upper leaflet states. These interaction energies estimate the enthalpic contribution to the free energy of binding, while the entropic contribution was not considered due to the high complexity in calculations. The enthalpic energy is an extensive thermodynamic quantity and its value is proportional to the mass of the peptide and the membrane system. Thus, the interaction energies are only used to show the relative difference of peptide-membrane/peptide-peptide interaction strength for the peptide among the three states: lower leaflet, the center and the upper leaflet states.

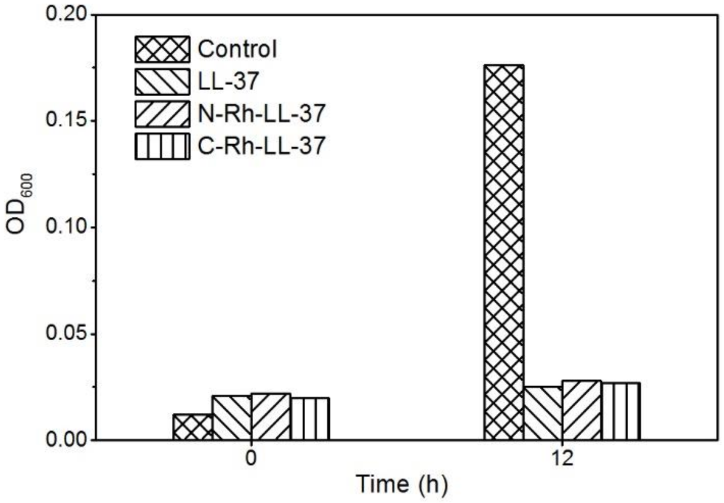

Supplementary Figure 10. The antimicrobial activity of 4 μM LL-37. We used E. coli to test the antimicrobial activity of the labeled LL-37. The cell growth was inhibited thoroughly at the concentration of 4 μM. The labeled LL-37 has nearly the same antimicrobial activity as the unlabeled one.

Supplementary Table 1. Maximum z-position of peptide centroid obtained from the 20 1-μs coarse-grained MD runs. z_{max} indicates the maximum z-position that the LL-37 peptides are able to reach. Note that at the initial state, z_{max} =1.38 nm and the DMPG bilayer is thinner than 4 nm.

MD Run	z_{max} (nm)	MD Run	z_{max} (nm)	MD Run	z_{max} (nm)	MD Run	z_{max} (nm)
1	1.46	6	1.38	11	1.62	16	1.64
2	1.66	7	**2.58**	12	1.38	17	1.46
3	1.68	8	1.40	13	**2.84**	18	1.69
4	1.40	9	1.56	14	1.66	19	**2.35**
5	**2.14**	10	1.38	15	**2.50**	20	1.38

Supplementary Table 2. Minimum z-position of peptide centroid obtained from the 20 1-μs coarse-grained MD runs. z_{min} indicates the minimum z-position that the LL-37 peptides are able to reach.

MD Run	z_{min} (nm)	MD Run	z_{min} (nm)	MD Run	z_{min} (nm)	MD Run	z_{min} (nm)
1	-1.04	6	**-2.29**	11	-1.22	16	-1.54
2	-1.53	7	-1.13	12	-1.53	17	-0.80
3	**-2.22**	8	-1.68	13	-1.26	18	-1.40
4	-1.20	9	-1.00	14	**-2.42**	19	-1.75
5	-1.83	10	-1.08	15	**-1.98**	20	-0.70

Supplementary Methods

Details of the atomistic MD simulations for the LL-37 monomer system.

We performed four independent 100-ns atomistic MD simulations started from a configuration where the LL-37 monomer was partially inserted in DMPG lipid bilayer. Due to the very long simulation time scales required to capture insertion events at physiological temperatures, computational studies of spontaneous insertion of peptide into atomistic bilayers are mostly unfeasible[1]. We therefore chose the membrane-bound helical LL-37 with the N-terminal residues 1-15 pre-inserted inside the upper leaflet of a DMPG lipid bilayer as the starting state of MD simulations, as done previously by us[2,3] and others[4] for other peptides. This strategy would allow us to examine whether LL-37 preferentially binds to the membrane surface or stays inside the bilayer within the time scales accessible in atomistic molecular dynamics simulations. The reason that we selected the initial state with the N-terminal residues pre-inserted in the lipid bilayer is that previous experimental studies reported that the N-terminal residues are involved in the membrane entry of LL-37[5-7].

All of the MD simulations were carried out in the isothermal-isobaric (NPT) ensemble with GROMACS 4.5.3 software package[8]. The LL-37 peptide was described using GROMOS87 force field[9]. The force-field parameters of DMPG were based on Berger et al.[10], with those for the glycerol group taken from Elmore[11], as done previously by us[2,3,12,13] and other groups[14-16]. To justify the use of the modified GROMOS87 force field here instead of more recent united-atom GROMOS96-based force fields, we performed four additional independent MD simulations using the GROMOS53A6/GROMOS-CKP force field[17].

The integration time step for MD simulations is 2 fs. Peptide bonds were constrained by the LINCS algorithm[18] and water geometries were constrained by SETTLE method[19]. The pressure was maintained at 1 bar using a semi-isotropic scheme in which the lateral and perpendicular pressures were coupled separately with a coupling constant of 1.0 ps and a compressibility of 4.5 $\times 10^{-5}$ bar^{-1} using isotropic Parrinello-Rahman's method[20,21]. The temperature was maintained at 310 K using Nose-Hoover's method[22,23]. Long-range electrostatic interaction was calculated using the Particle Mesh Ewald (PME) method[24] with a real space cutoff of 1.2 nm, as recommended for membrane simulations, especially for those involving charged lipids. The van der Waals

interaction was calculated using a cutoff of 1.4 nm.

Details of the coarse-grained MD simulations.

We performed twenty 1-μs coarse-grained (CG) MD simulations for the 8-mer LL-37 toroidal pore and four 1-μs CG-MD simulations for the 10-mer pore in DMPG lipid bilayer. All MD simulations were carried out in the isothermal-isobaric (NPT) ensemble using GROMACS 4.5.3 software package[8]. We used the MARTINI coarse-grained model[25-27] to simulate the lipids, amino acids and water molecules, as done in previous studies on membrane proteins[28-33]. This force field allows a 4-fold reduction in the number of particles represented and a 10~30-fold increase in the time step size in MD, as compared with united-atom simulations[25]. The MD integration time step is 20 fs[34]. The time scales quoted in this work are real simulated time, and should be scaled by a factor of four to correct for the faster diffusion rates of water and lipids in the coarse-grained model[25]. The system is weakly coupled to external temperature and pressure baths using the Berendsen coupling methods[35]. A temperature of 310 K is kept with a coupling constant of 0.3 ps, above the gel-liquid crystal phase transition temperature ~300 K of DMPG lipid bilayers[36]; a semi-isotropic pressure of 1 atm is maintained with a coupling constant of 3 ps. The vdW potential is shifted from 0.9 to 1.2 nm, and the electrostatic potential is shifted from 0.0 to 1.2 nm[26]. The dielectric constant in the simulations is ε_r = 2.5. The neighbor list is updated every 10 steps with a cutoff distance of 1.2 nm.

Coarse-grained model of an LL-37 peptide and a DMPG lipid molecule.

The NMR structure[5] of LL-37 in micelles by Wang (PDB ID: 2K6O) is used to generate the coarse-grained model, in which the peptide mainly adopts predominantly α-helical structure for residues 2-31 with a kink of residues 14-16 and disordered structure in C-terminal tail. As secondary structure changes of proteins cannot be modeled in Martini model, to initiate a simulation using MARTINI model, the secondary structure should be first assigned to the peptide chains and the assigned secondary structure remains fixed during the simulation[26]. At neural pH, the side chains of Arg and Lys are positively charged, while the side chains of Asp and Glu are negatively charged. The glycerol group is modeled by one polar bead (type P4), the phosphate group by a bead with a charge of -1 (type Qa), the ester linkage by two intermediate polar beads

(type Na), and the saturated fatty acid by three apolar beads (type C1) each tail[25]. Thus a DMPG lipid has one negative net charge.

System setup for LL-37 toroidal pores for the coarse-grained MD simulations.

Neutron in-plane scattering experimental studies reported that the water channel radius of the transmembrane pore formed by LL-37 is ~3.3 nm at the peptide/lipid molar ratio of 1/50.[37] As previous studies suggest LL-37 forms a toroidal pore[37-40], an 8-membered pore model (inner radius≈3.2 nm) is built with the hydrophilic face of LL-37 exposed to water solution, and placed into the DMPG bilayer by the program INFLATEGRO from Tieleman's group[41] with lipids inside the pore removed. Counterions (Na+) are added to neutralize the system. This system is energy minimized and solvated with water, followed by energy minimization and a protein-position-restrained simulation for 100 ns, to obtain a well-equilibrated toroidal pore. In this work, the peptide/lipid ratio is 8/458, above the experimentally-determined peptide/lipid molar ratio of 1/50 for transmembrane pore formation[37]. We also carry out MD simulations in other peptide/lipid ratios, and find that a stable toroidal structure cannot exist when the number of peptides is less than six.

System setup for LL-37 toroidal pores for the atomistic MD simulation.

We also performed one 350-ns atomistic MD simulation in order to probe the structural stability of a more realistic LL-37 pore in DMPG bilayer. This simulation started from an octamer in which each of the eight peptide chain is in an α-helical structure and perpendicular to the normal of the DMPG bilayer. The LL-37 peptide and the DMPG lipids are described respectively using the GROMOS87 force field[9] and the modified Berger parameters[10,11]. Similar to the CG-MD simulations, the peptide/lipid ratio is 8/458. There are 127,509 atoms in this atomistic LL-37 octamer-membrane system. It took about 2 months for a 350-ns MD simulation using 96-cores on a PC-cluster. It can be seen from Supplementary Figure 8 that the LL-37 pore remained stable during the full period MD simulation and it gradually changed into a toroidal shape. In addition, each LL-37 peptide kept α-helical conformation.

References

1 Jaud, S. *et al.* Insertion of short transmembrane helices by the Sec61 translocon. *Proc. Natl. Acad. Sci. U.S.A.* **106**, 11588-11593 (2009).

2 Chang, Z., Luo, Y., Zhang, Y. & Wei, G. Interactions of Abeta25-35 beta-barrel-like oligomers with anionic lipid bilayer and resulting membrane leakage: an all-atom molecular dynamics study. *J. Phys. Chem. B* **115**, 1165-1174 (2011).

3 Zhang, Y., Luo, Y., Deng, Y., Mu, Y. & Wei, G. Lipid interaction and membrane perturbation of human islet amyloid polypeptide monomer and dimer by molecular dynamics simulations. *PloS one* **7**, e38191 (2012).

4 Soliman, W., Bhattacharjee, S. & Kaur, K. Interaction of an antimicrobial peptide with a model lipid bilayer using molecular dynamics simulation. *Langmuir* **25**, 6591-6595 (2009).

5 Wang, G. Structures of human host defense cathelicidin LL-37 and its smallest antimicrobial peptide KR-12 in lipid micelles. *J. Biol. Chem.* **283**, 32637-32643 (2008).

6 Thennarasu, S. *et al.* Antimicrobial and membrane disrupting activities of a peptide derived from the human cathelicidin antimicrobial peptide LL37. *Biophys. J.* **98**, 248-257 (2010).

7 Johansson, J., Gudmundsson, G. H., Rottenberg, M. n. E., Berndt, K. D. & Agerberth, B. Conformation-dependent antibacterial activity of the naturally occurring human peptide LL-37. *J. Biol. Chem.* **273**, 3718-3724 (1998).

8 Van Der Spoel, D. *et al.* GROMACS: fast, flexible, and free. *J. Comput. Chem.* **26**, 1701-1718 (2005).

9 Van Gunsteren, W. & Berendsen, H. *Gromos-87 Manual*. (The Netherlands, 1987).

10 Berger, O., Edholm, O. & Jahnig, F. Molecular dynamics simulations of a fluid bilayer of dipalmitoylphosphatidylcholine at full hydration, constant pressure, and constant temperature. *Biophys. J.* **72**, 2002-2013 (1997).

11 Elmore, D. E. Molecular dynamics simulation of a phosphatidylglycerol membrane. *FEBS Lett.* **580**, 144-148 (2006).

12 Guo, C., Cote, S., Mousseau, N. & Wei, G. Distinct helix propensities and membrane interactions of human and rat IAPP(1-19) monomers in anionic lipid bilayers. *J. Phys. Chem. B* **119**, 3366-3376 (2015).

13 Qian, Z., Jia, Y. & Wei, G. Binding Orientations and Lipid Interactions of Human Amylin at Zwitterionic and Anionic Lipid Bilayers. *J. Diabetes Res.* **2016**, 1749196 (2016).

14 Dickey, A. & Faller, R. Examining the contributions of lipid shape and headgroup charge on bilayer behavior. *Biophys. J.* **95**, 2636-2646 (2008).

15 Yi, M., Nymeyer, H. & Zhou, H. X. Test of the Gouy-Chapman theory for a charged lipid membrane against explicit-solvent molecular dynamics simulations. *Phys. Rev. Lett.* **101**, 038103 (2008).

16 Broemstrup, T. & Reuter, N. Molecular dynamics simulations of mixed acidic/zwitterionic phospholipid bilayers. *Biophys. J.* **99**, 825-833 (2010).

17 Piggot, T. J., Holdbrook, D. A. & Khalid, S. Electroporation of the E. coli and S. Aureus

membranes: molecular dynamics simulations of complex bacterial membranes. *J. Phys. Chem. B* **115**, 13381-13388 (2011).

18 Hess, B., Bekker, H., Berendsen, H. J. C. & Fraaije, J. G. E. M. LINCS: A linear constraint solver for molecular simulations. *J. Comput. Chem.* **18**, 1463-1472 (1997).

19 Miyamoto, S. & Kollman, P. A. Settle: An analytical version of the SHAKE and RATTLE algorithm for rigid water models. *J. Comput. Chem.* **13**, 952-962 (1992).

20 Parrinello, M. & Rahman, A. Polymorphic transitions in single crystals: A new molecular dynamics method. *J. Appl. Phys.* **52**, 7182-7190 (1981).

21 Nosé, S. & Klein, M. L. Constant pressure molecular dynamics for molecular systems. *Mol. Phys.* **50**, 1055-1076 (1983).

22 Nosé, S. A molecular dynamics method for simulations in the canonical ensemble. *Mol. Phys.* **52**, 255-268 (1984).

23 Hoover, W. G. Canonical dynamics: Equilibrium phase-space distributions. *Phys. Rev. A* **31**, 1695-1697 (1985).

24 Essmann, U. *et al.* A smooth particle mesh Ewald method. *The Journal of Chemical Physics* **103**, 8577-8593 (1995).

25 Marrink, S. J., Risselada, H. J., Yefimov, S., Tieleman, D. P. & de Vries, A. H. The MARTINI force field: coarse grained model for biomolecular simulations. *J. Phys. Chem. B* **111**, 7812-7824 (2007).

26 Monticelli, L. *et al.* The MARTINI coarse-grained force field: extension to proteins. *J. Chem. Theory Comput.* **4**, 819-834 (2008).

27 Yesylevskyy, S. O., Schäfer, L. V., Sengupta, D. & Marrink, S. J. Polarizable water model for the coarse-grained MARTINI force field. *PLoS Comput. Biol.* **6**, e1000810 (2010).

28 Louhivuori, M., Risselada, H. J., van der Giessen, E. & Marrink, S. J. Release of content through mechano-sensitive gates in pressurized liposomes. *Proc. Natl. Acad. Sci. U.S.A.* **107**, 19856-19860 (2010).

29 van den Bogaart, G. *et al.* Membrane protein sequestering by ionic protein-lipid interactions. *Nature* **479**, 552-555 (2011).

30 Fuhrmans, M. & Marrink, S. J. Molecular view of the role of fusion peptides in promoting positive membrane curvature. *J. Am. Chem. Soc.* **134**, 1543-1552 (2012).

31 Arnarez, C., Mazat, J.-P., Elezgaray, J., Marrink, S.-J. & Periole, X. Evidence for cardiolipin binding sites on the membrane-exposed surface of the cytochrome bc1. *J. Am. Chem. Soc.* **135**, 3112-3120 (2013).

32 Konijnenberg, A. *et al.* Global structural changes of an ion channel during its gating are followed by ion mobility mass spectrometry. *Proc. Natl. Acad. Sci. U.S.A.* **111**, 17170-17175 (2014).

33 Santo, K. P. & Berkowitz, M. L. Difference between magainin-2 and melittin assemblies in phosphatidylcholine bilayers: Results from coarse-grained simulations. *J. Phys. Chem. B* **116**, 3021-3030 (2012).

34 Marrink, S. J., Periole, X., Tieleman, D. P. & de Vries, A. H. Comment on "On using a too large integration time step in molecular dynamics simulations of coarse-grained molecular models" by M. Winger, D. Trzesniak, R. Baron and W. F. van Gunsteren, Phys. Chem. Chem. Phys., 2009, 11, 1934. *Phys. Chem. Chem. Phys.* **12**, 2254-2256 (2010).

35 Berendsen, H. J. C., Postma, J. P. M., van Gunsteren, W. F., DiNola, A. & Haak, J. R. Molecular

dynamics with coupling to an external bath. *J. Chem. Phys.* **81**, 3684-3690 (1984).
36 Lewis, R. N. A. H., Zhang, Y.-P. & McElhaney, R. N. Calorimetric and spectroscopic studies of the phase behavior and organization of lipid bilayer model membranes composed of binary mixtures of dimyristoylphosphatidylcholine and dimyristoylphosphatidylglycerol. *Biochim. Biophys. Acta* **1668**, 203-214 (2005).
37 Lee, C.-C., Sun, Y., Qian, S. & Huang, Huey W. Transmembrane pores formed by human antimicrobial peptide LL-37. *Biophys. J.* **100**, 1688-1696 (2011).
38 Henzler Wildman, K. A., Lee, D.-K. & Ramamoorthy, A. Mechanism of lipid bilayer disruption by the human antimicrobial peptide, LL-37. *Biochemistry* **42**, 6545-6558 (2003).
39 Brogden, K. A. Antimicrobial peptides: pore formers or metabolic inhibitors in bacteria? *Nat. Rev. Micro.* **3**, 238-250 (2005).
40 Gable, J. E., Schlamadinger, D. E., Cogen, A. L., Gallo, R. L. & Kim, J. E. Fluorescence and UV resonance Raman study of peptide−vesicle interactions of human cathelicidin LL-37 and its F6W and F17W mutants. *Biochemistry* **48**, 11264-11272 (2009).
41 Kandt, C., Ash, W. L. & Peter Tieleman, D. Setting up and running molecular dynamics simulations of membrane proteins. *Methods* **41**, 475-488 (2007).
